# How the mechanical microenvironment of stem cell growth affects their differentiation: a review

**DOI:** 10.1186/s13287-022-03070-0

**Published:** 2022-08-13

**Authors:** Xiaofang Zhang, Sibo Zhang, Tianlu Wang

**Affiliations:** 1 Department of Radiotherapy, Cancer Hospital of China Medical University, Liaoning Cancer Hospital and Institute, Shenyang, China; 2 China Medical University, Shenyang, China

**Keywords:** Stem cell, Extracellular matrix, Shear stress, Hydrostatic pressure, Tension, Microgravity, NF-kB, nAChR, PIEZO, HIF-1α

## Abstract

Stem cell differentiation is of great interest in medical research; however, specifically and effectively regulating stem cell differentiation is still a challenge. In addition to chemical factors, physical signals are an important component of the stem cell ecotone. The mechanical microenvironment of stem cells has a huge role in stem cell differentiation. Herein, we describe the knowledge accumulated to date on the mechanical environment in which stem cells exist, which consists of various factors, including the extracellular matrix and topology, substrate stiffness, shear stress, hydrostatic pressure, tension, and microgravity. We then detail the currently known signalling pathways that stem cells use to perceive the mechanical environment, including those involving nuclear factor-kB, the nicotinic acetylcholine receptor, the piezoelectric mechanosensitive ion channel, and hypoxia-inducible factor 1α. Using this information in clinical settings to treat diseases is the goal of this research, and we describe the progress that has been made. In this review, we examined the effects of mechanical factors in the stem cell growth microenvironment on stem cell differentiation, how mechanical signals are transmitted to and function within the cell, and the influence of mechanical factors on the use of stem cells in clinical applications.

## Background

Stem cells have unquestionable importance in medicine and are receiving increasing attention due to their role in several diseases [1, 2]. Stem cell renewal [3], migration [4], adhesion [5], and differentiation [6] are integral to the proper functioning of living organisms, and their dysregulation can lead to multiple diseases. The majority of research on stem cell differentiation concerns how chemical stimulation affects differentiation [7]. However, there is growing evidence of the importance of physical signals in stem cell fate [8]. The application of specific physical factors can propel the differentiation of stem cells in a more clinically favourable and specialized direction [9–11].

The microenvironment in which stem cells grow contains not only a variety of biochemical molecules, but also a variety of mechanical factors [12]. The mechanical microenvironment of stem cells alters the fate of stem cells [13] and regulates multiple signalling cascade responses involved in stem cell differentiation [14]. And different types of stem cells will differentiate in different directions and show different fates when responding to the same microenvironmental signals. The effect of these mechanical signals on stem cell differentiation has an important role in clinical application [15] (Table 1).

**Table 1:** Role and application of mechanical signals on stem cell differentiation

Physical signal	Mechanical signal	Responsive cell	Effectiveness of mechanical signal	Application	References
ECM	dECM	BMSC	Enhancing osteogenic and angiogenic potential	Optimization of cell culture conditions	[20]
	3D Microenvironment	hESC	Promoting gene expression associated with differentiation to neural crest stem cells and osteoblasts	Optimizing artificial scaffolds as culture conditions	[23]
	ECM and artificial scaffolds	hASC	Corresponding cell-derived ECM promotes corresponding differentiation	Improving the regenerative capacity of unmodified scaffolds	[26]
Substrate topology	Low pore size fibres	hMSC	Enhancing osteogenesis	Inducing stem cell-directed differentiation	[32]
	Large pore size fibres	rAMSC	Promoting differentiation into islet-like clusters		[33]
	Porous topology	NSPC	Promoting differentiation into astrocytes and neurons		[34]
	Composite microstructure of nanofibres	rBMSC	Enhancing osteogenic differentiation		[35]
Substrate hardness	High hardness 3D-printed ECM	BMSC	Differentiating into sweat gland cells and hair follicle cells		[38]
	Hard alginate shells	hMSC	Promoting osteogenic differentiation		[39]
	Soft hydrogel	VPC	Inducing differentiation towards endothelial cells		[40]
Shear stress	Oscillatory shear stress	rBMSC	Promoting osteogenic differentiation	Bone tissue engineering	[48]
	Intermittent shear stress	rBMSC	Enhancing osteogenic differentiation		[49]
	Perfusion culture	3D MT-dASC	Changing in direction of osteogenic differentiation to lipogenic differentiation		[55]
Hydrostatic pressure	Circulating hydrostatic pressure	MSC	Enhancing osteogenic response	Changing the direction of stem cell differentiation	[59]
	Circulating hydrostatic pressure and decalcified bone matrix scaffold	MSC	Reducing osteogenic properties and enhancing chondrogenic properties		[60]
Tension	Cyclic mechanical draft force	Human periodontal stem cells	Promoting osteogenic differentiation	Dental tissue engineering	[61–63]
	Cyclic stretching	EPCs	Differentiating towards endothelium and angiogenesis	Vascular regeneration project	[64]
		Bone marrow-derived cells	Expressing smooth muscle cell markers		[65]
Microgravity	Microgravity	hBMSC	Inhibiting osteogenic differentiation and promoting adipogenic and chondrogenic differentiation	Treatment of diseases related to bone loss in space	[75, 76]
	Nanostands and microgravity	hBMSC	Mitigating microgravity-induced osteoblast dysfunction		[77]
	Simulation of microgravity	mESC	Differentiating towards the stereotyped endoderm	Contribution to the study of regeneration engineering	[79]

*ECM* extracellular matrix, *BMSCs* bone marrow mesenchymal stem cells (MSCs), *hESCs* human embryonic stem cells, *hASCs* human adipose stem cells, *hMSC* human MSC, *rAMSCs* rat adipose MSCs, *VPC* vascular progenitor cell, *rBMSC* rat bone marrow MSC, *3D MT-dASC* 3D microtissue-derived adult stem cell, *mESC* mouse embryonic stem cell, *NSPC* neural stem progenitor cell, *EPCs* endothelial progenitor cells

The cytoskeleton plays an indispensable role in the cell’s perception of the mechanical environment. Actin senses the stiffness of the environment and controls the persistence of the platelet foot through a specific cluster of actin/proto-myosin filaments, which in turn assembles a focal adhesion [16]. The focal adhesion then binds to extracellular matrix (ECM) ligands as well as intracellular proteins [17]. The mechanical signal is then transmitted to the nucleus via stress fibres [14]. Fibre stiffness in turn acts on ligand density at the cell surface and promotes the formation of the focal adhesion and associated signalling [18]. In addition to the transmission of perceived mechanical signals through the cytoskeleton to regulate stem cell differentiation, cells also transform mechanical signals into cell-recognizable chemical signals through various mechanosensors and transduction mechanisms [17], which in turn regulate stem cell differentiation.

This review aims to summarize the impact of mechanical factors in the stem cell growth microenvironment on stem cell differentiation, describe how stem cells sense and respond to mechanical signals to function, and further explore the clinical implications of the influence of mechanical factors on stem cells.

## Mechanical microenvironment for stem cell growth

The stem cell microenvironment refers to all the environmental factors surrounding stem cells in tissues as they proliferate, self-renew, and differentiate into tissue cells at their residency sites, including soluble biomolecules, solid ECM with supporting cells, and the mechanical and physicochemical environment surrounding the stem cells. The mechanical microenvironment is an integral part of the stem cell microenvironment and includes the mechanical support of the ECM, the forces exerted on the cell by the environment, and the forces caused by the interaction of the cell with the surrounding support cells [19]. In vivo, the development, growth, proliferation, and differentiation of stem cells are inseparable from the mechanical environment in which they are embedded. In the usual physiological mechanical environment, stem cells can perform their functions normally; when the surrounding environment changes, the various functions of stem cells also change. Multiple aspects of the influence of the mechanical microenvironment of stem cell growth on their differentiation can be studied, including the extracellular matrix, substrate topology, substrate stiffness, shear stress, tension, hydrostatic pressure, and microgravity.

### Extracellular matrix and topology

ECM is a three-dimensional network of various extracellular macromolecules, such as collagen, elastin, fibronectin, and laminin proteins, which provide a good environment for cells to survive. Cells cultured in vitro secrete ECM and the cell-derived ECM (dECM) produced after cell removal has great application in cell culture. For example, culturing bone marrow mesenchymal stem cell (MSC) (BMSC) with different combinations of distinct cell types of dECM showed that dECM enhanced the osteogenic and angiogenic potential of BMSC compared to tissue culture polystyrene; the different behaviour of the BMSC is related to the different proportions of cells that make up the dECM [20]. In addition, the natural living environment of cells is three-dimensional and 3D culture better simulates the realistic living environment of the cells. When cells are cultured in 3D, their biological behaviour and morphological size are altered, thus changing their surrounding mechanical microenvironment. Morphological changes and geometry of cells can modulate nanostructures and lipid assembly within cell membranes, thereby regulating stem cell signalling and differentiation fate [21]. The study of 3D materials will contribute to the development of tissue engineering and regenerative medicine [22]. Artificial scaffolds can be used to replace natural ECM as a cell culture medium. β-tricalcium phosphate (β-TCP) scaffolds with ECM-like properties provide a 3D microenvironment for human embryonic stem cell (hESC) and promote the expression of genes associated with neural crest stem cells and osteoblast differentiation [23]. Therefore, the continuous optimization of artificial scaffolds as culture conditions will help to further explore how ECM affects stem cell differentiation. Osteogenic differentiation of stem cells was significantly increased when 3D scaffolds were combined with heparin and bone morphogenetic protein 2 (BMP-2) [24]. Osteogenic differentiation was also significantly enhanced in MSCs growing on alginate/graphene oxide-printed 3D scaffolds [25]. Other groups have attempted to combine artificial stents with ECM, combining cell-derived ECM with 3D-printed polycaprolactone (PCL) scaffolds to culture human adipose stem cells (ASCs) (hASCs). Chondrocyte-derived ECM promoted cartilage differentiation and osteoblast-derived ECM was able to stimulate hASCs towards osteogenic differentiation [26]. Cell-derived ECM therefore has great potential to enhance the regenerative capacity of unmodified PCL stents and warrants further study, offering the possibility of determining the fate of stem cell differentiation. Interestingly, the effects of the 3D environment on stem cells are not static, but diverse. Induced pluripotent stem cell (iPSC) differentiates into a typical MSC-like phenotype on tissue culture plastic or on the surface of fibrin hydrogels. In contrast, iPSCs embedded in a 3D environment do not differentiate towards MSC and have reduced differentiation potential for osteogenic and lipogenic lineages [27].

The topology of the substrate is a physical characteristic of the microenvironment in which the cells are anchored and affects stem cell differentiation in many ways. Nanoscale materials have been widely used to model ECM and their topology has a significant impact on the fate of stem cells [28, 29]. For example, Jaswal et al. investigated electrospun nanofibre scaffolds for peripheral nerve regeneration [30] and found that precisely controlled concentrations of reduced graphene-encapsulated gold nanoparticles in PCL fibre scaffolds provided a microenvironment that mimicked natural ECM, and that their uniformly distributed topology might increase the stimulation of cell differentiation and could promote neuronal network formation. In addition, the nanotopography enhances the hydrophilicity of 3D-printed polylactic acid (PLA) scaffolds and significantly enhances osteogenic differentiation on the scaffold [31]. The study of how scaffold topology regulates cell behaviour can be considered from several perspectives, including pore size, porosity, fibre morphology, fibre diameter, and orientation. When human MSC (hMSC) were inoculated in 3D ECM-like fibrous structures, the smaller pore size exhibited higher overall stiffness and significantly enhanced hMSC collagen and mineral deposition, enhancing osteogenesis [32]. A 3D electrospun nanofibre scaffold with a large pore size supported the differentiation of rat adipose MSCs (rAMSCs) into islet-like clusters [33]. The addition of different copolymers to PCL produced micron fibres with a porous topology that allowed cultured rat neural stem progenitor cell (NSPC) to differentiate into astrocytes and neurons in the absence of any growth factors, demonstrating the role of the porous topology of the fibres [34]. Electrostatic spinning scaffolds of calcium phosphate nanoparticles with a composite microstructure of microbeads and nanofibres can enhance the osteogenic differentiation of rBMSCs by promoting scaffold biomineralization and protein adsorption through the exposure of bioactive components [35], which has potential in bone regeneration. The highest expression of insulin-differentiated cells was found on 300 nm-diameter fibres when mouse ESCs were cultured in a reticulated fibrous medium formed from polyamide (PA) fibres [36].

### Substrate stiffness

Under physiological conditions, cells in vivo are anchored to tissue substrates of varying stiffness, and their specific stiffness influences the cells that grow on them. Substrate stiffness has a role in a wide range of stem cell differentiation profiles [37]. Cells perceive the stiffness of ECM through cytoskeletal contractility, and the relatively high stiffness of 3D-printed ECM facilitates the differentiation of BMSCs towards sweat cells and hair follicle cells [38]. The harder alginate shell promotes osteogenic differentiation of hMSCs [39], whereas the softer hydrogel will direct the differentiation of vascular progenitor cells (VPCs) towards endothelial cells (ECs) [40]. In addition, stiffness and topology have a synergistic effect on the maintenance of stem cell characteristics and the adipogenic or osteogenic differentiation of mouse MSCs (mMSCs) [41]. Matrix stiffness plays a dominant role in the maintenance of stemness on hard gels and hepatic differentiation on soft gels, whereas matrix morphology contributes to hepatocyte-like differentiation on soft gels [42]. MSC can interestingly no longer perceive the difference between soft and hard substrates after a period of incubation on a rigid substrate [43]. Therefore, in addition to the synergistic effect of substrate morphology and substrate hardness, incubation time also plays a role in regulating the differentiation of MSCs. The response of MSCs to substrate morphology varies depending on substrate stiffness and incubation time, and the effect of substrate stiffness and incubation time on MSCs also depends on the morphology of the substrate arrangement [44].

### Shear stress

All types of tissue cells in the normal human body are continuously exposed to shear stresses caused by fluid flow in the tissue interstices under load. Shear force treatment of hESC simulated by the Microfluidic Dynamic Culture System promotes the expression of blood progenitors in the hESC lineage, reducing the proportion of mono-competent erythroid and megakaryocyte lineages and increasing the number of bone marrow and bipotent megakaryocyte–erythroid progenitors. Shear force treatment also promoted smooth muscle and cardiomyocyte production, suggesting a role for shear stress in both the haematopoietic spectrum and the arterial vascular system [45]. Human pluripotent stem cell-derived endothelial cells (hPSC-ECs) are more sensitive to low levels of shear stress and require prolonged exposure to shear stress to trigger stable phenotypic changes, exhibiting increased expression of arterial markers, suggesting that hPSC-ECs are transformed into an arterial phenotype [46]. Thus, shear stress influences the differentiation of stem cells.

Perfusion flow-induced shear stress in a fully automated bioreactor enhances osteogenic differentiation in hBMSC and modulates O_2_ concentration to improve osteogenic differentiation. This bioreactor is used to precisely control the fate of stem cells in terms of osteogenesis and has potential applications in the healthcare industry, for example in the prevention of osteoporosis [47]. However, although shear stress induced by perfusion flow was demonstrated to have a role in inducing stem cell differentiation, different patterns, sizes, and rates of fluid shear stimulation have different effects on stem cells. Osteogenic differentiation of rat bone marrow MSCs (rBMSCs) is more strongly promoted by 0.0225 Pa oscillatory shear stress [48]. Intermittent shear stresses of the order of 10 mPa can effectively enhance the osteogenic differentiation of rBMSCs [49]. The rate of fluid shear stress can also control the fate of rBMSCs towards osteogenic or chondrogenic cell differentiation [50]. The effect of osteogenic differentiation of rBMSCs under different shear stresses could be useful for bone tissue engineering applications. Therefore, the study of different stresses on the induction of osteogenic differentiation of stem cells has great importance.

However, a single application of shear stress is not sufficient. In practice, a combination of other culture conditions should be considered to improve the efficiency of targeted differentiation. Shear stress combined with polymeric biomaterials can enhance the osteogenic differentiation of MSCs [51]. This combination has driven improvements in the clinical approach to treating bone defects. High levels of cardiac-related gene expression were not observed in either of the 5-azacytidiner (5-Aza) or shear stress groups, whereas BMSCs cultured with 5-Aza in concert with shear stress showed significantly increased cardiac-related gene expression [52], which is expected to promote cardiac differentiation of stem cells. In addition to shear stress combining with biochemical conditions to regulate stem cell behaviour, shear stress can also work in concert with other physical conditions. Shear-stressed groove structures can promote the differentiation of BMSCs into myofibroblasts [53]. The hBMSCs embedded in the 3D scaffold are subjected to shear stress to produce a typical tendonogenic phenotype and promote the expression of tendon gene markers [54]. Osteogenic differentiation of 3D microtissue-derived human stem cells on bone bionic electrospun nanocomposites was evident, but shear stress led to lipogenic differentiation of 3D microtissue-derived human stem cells under perfusion culture [55]. This result not only links the 3D environment and composite materials to stem cell differentiation, but also contributes to changing the direction of stem cell differentiation.

### Hydrostatic pressure

Hydrostatic pressure (HP) is a mechanical force that is widely present in the environment in which cells live. Using autologous platelet-rich fibrin (PRF) membranes as a growth factor-rich scaffold and culturing BMSCs pre-conditioned with HP prior to transplantation greatly enhanced the chondrogenic potential of the BMSC/PRF constructs. Further studies showed that the pressurized pretreated BMSC/PRF graft group showed a significant improvement in the integration of the regenerated cartilage with the host cartilage environment [56]. HP is therefore worth considering in the application of stem cell differentiation.

HP combined with the stromal microenvironment can induce directed differentiation of stem cells. Intermittent HP (IHP) and 3D microenvironments modified with ECM proteins, especially collagen, have a synergistic effect on the expression of chondrogenic genes by MSCs [57]. HP and piezoelectric scaffolds also have a synergistic effect on promoting chondrogenic differentiation in MSCs [58]. Circulating HP (CHP) increases the MSC osteogenic response through cytoskeletal reorganization [59]. However, when CHP was applied to hBMSCs in a decalcified bone matrix (DBM) scaffold, it reduced osteogenic properties and favoured chondrogenic properties [60], suggesting that HP combined with different induction conditions could alter the direction of stem cell differentiation.

### Tension

Tension is the force exerted on an object by means of pulling in a certain direction, such as the pulling force produced on the cells in the body by the tissues. For example, muscle contraction is a movement produced by the cells being subjected to traction. Multiple studies confirm that cyclic mechanical tension can promote osteogenic differentiation of human periodontal stem cells [61–63]. Further studies have elucidated the contribution of tensile forces to stem cell differentiation. Circulating stretch not only promotes endothelial differentiation and angiogenesis of endothelial progenitor cells (EPCs) [64], but also enhances the expression of smooth muscle cell markers by bone marrow-derived cells [65], which has applications in vascular regeneration engineering. Appropriate tensile strain promotes osteogenic differentiation of BMSCs while inhibiting differentiation to adipocytes [66], and uniaxial cyclic stretch is even more significant in inducing MSC differentiation to osteoblasts in vitro [67]. This result also suggests that different types of tension act in different ways on stem cells.

In addition, the frequency and amplitude as well as the duration of stretching led to the differentiation of cells in different directions [68]. For example, the gene expression of type I collagen (Col I) and glycosaminoglycan (GAG) was significantly upregulated in the 10% and 15% stretch groups, whereas the gene expression of type II collagen (Col II) was downregulated, leading to differentiation towards fibrochondrocytes. However, a higher stretch stimulus (15%) simultaneously promoted the synthesis of α-smooth muscle actin. Therefore, 10% radial stretch stimulation is the optimal intensity to induce differentiation of BMSCs into fibrochondrocytes [69].

### Microgravity

A microgravity environment is one in which the apparent weight of a system is much less than its actual weight in the presence of gravity. Microgravity is not common in daily life, but it has a major impact on astronauts conducting space operations. Microgravity can up- or downregulate differentiation-related genes [70, 71], which may lead to a range of related disorders in astronauts, such as bone loss [72] and cardiovascular disease [73]. In addition, simulated microgravity conditions may also disrupt the homeostasis of the immune system and lead to dynamic changes in hematopoietic stem cells (HSC) and lineage cells [74]. These results contribute to a better understanding of immune regulation and its changes during spaceflight, thus providing possible directions for the prevention or treatment of immune system disorders in astronauts.

Many studies have demonstrated that microgravity inhibits the osteogenic differentiation of hBMSCs while promoting adipogenesis and chondrogenic differentiation [75, 76]. However, growth on a nanocrystalline magnesium-doped hydroxyapatite/type I collagen composite scaffold (MHA/Coll) can attenuate microgravity-induced osteoblast dysfunction in hBMSCs and promote cell differentiation along the osteogenic lineage [77]. In addition, nanocomplexes loaded with BMP2 and BMP7 in simulated microgravity can also promote osteogenic differentiation of human adipose-derived stem cells (hADSCs) [78]. These results indicate the possibility of treating diseases associated with bone loss in space.

Microgravity has a positive effect in some ways. Mouse embryonic stem cell (mESC) cultured in a rotating bioreactor under simulated microgravity conditions can differentiate towards stereotyped endoderm, and these cells can further differentiate into cells from other related organs such as the pancreas, liver, and thyroid [79]. Simulated microgravity also promoted the proliferation and matrix production of tissue-engineered human chondrocyte-like cells [80]. MSCs cultured with a liver induction medium are more conducive to liver differentiation under long-term microgravity conditions [81]. Microgravity is therefore useful for regenerative engineering studies and has potential applications for disease prevention and treatment.

## Mechanisms for perceiving the mechanical environment

As described in the previous section, mechanical stimuli, or the mechanical properties of the pericellular matrix material, play an important role in regulating the morphological development and function of stem cells. However, the exact mechanisms of how mechanical signals are sensed by and transmitted to and within stem cells, ultimately leading to a range of biological effects in stem cells, need to be further explored. There are two main mechanisms regarding the cellular perception of mechanical signals: transmission and transduction mechanisms. Signal transmission mechanisms occur when changes in the microenvironment are transmitted via sensors into the cell to cause rearrangement of the cytoskeleton, which in turn transmits signals to the cytoskeleton in the nucleus. Signal transduction mechanisms occur when changes in the microenvironment alter the permeability of ion channels or the activity of associated intracellular receptors, transducing mechanical signals into chemical signals to regulate the expression of associated genes. The two mechanisms work in synergy to transmit and convert mechanical signals. The cytoskeleton, integrins, and ion channels, among others, play important roles in perception [40]. In addition, various cellular sensory transduction pathways have been associated with mechanical stimulation, including signalling pathways such as NF-κB, nAChR, PIEZO, and HIF-1α.

### NF-κB signalling pathway

Nuclear factor κ-B (NF-κB) is a ubiquitous, inducible nuclear transcriptional activator that binds to enhancer elements in many different cell types and can also be activated by pathogenic stimuli. RANKL is a ligand for NF-κB receptor activator (RANK), which binds specifically to and activates RANK. RANKL-stimulated cells exhibit marked translocation of p65 from the cytoplasm to the nucleus, phosphorylating and activating NF-κB pathway-associated proteins. Activated NF-κB/p65 translocation to the nucleus reduces the expression of runt-related transcription factor 2 (RUNX2), alkaline phosphatase (ALP), and osteoprotegerin (OPG). At the same time, OPG is a decoy receptor for RANKL, which competitively inhibits the binding of RANKL to RANK, inhibiting osteoclast production and its survival time. Therefore, the relative ratio of RANKL/OPG, which coordinates osteoblast/osteoclast production, is of great importance [82].

Binding of NF-κB dimers to NF-κB inhibitor (IκB) in the cytoplasm renders NF-κB dimers inactive. IκB proteins are phosphorylated and degraded following physical or chemical stimulation of cells. Subsequently, NF-κB dimers are transformed into an activated state, released, and transferred to the nucleus, leading to nuclear localization of p65 and increased expression of RANKL mRNA, resulting in an increased RANKL/OPG ratio, which in turn induces target gene transcription [83]. Mechanical stretching reduces phosphorylated IκB kinase and IκBα degradation is inhibited, resulting in increased IκBα, reduced phosphorylation and nuclear accumulation of P65, and downregulated activity, which in turn blocks NF-κB activity and promotes osteogenesis in hBMSCs [84]. The NF-κB signalling pathway provides a starting point for more precise regulation of stem cell differentiation at the molecular level.

**Fig. 1:**
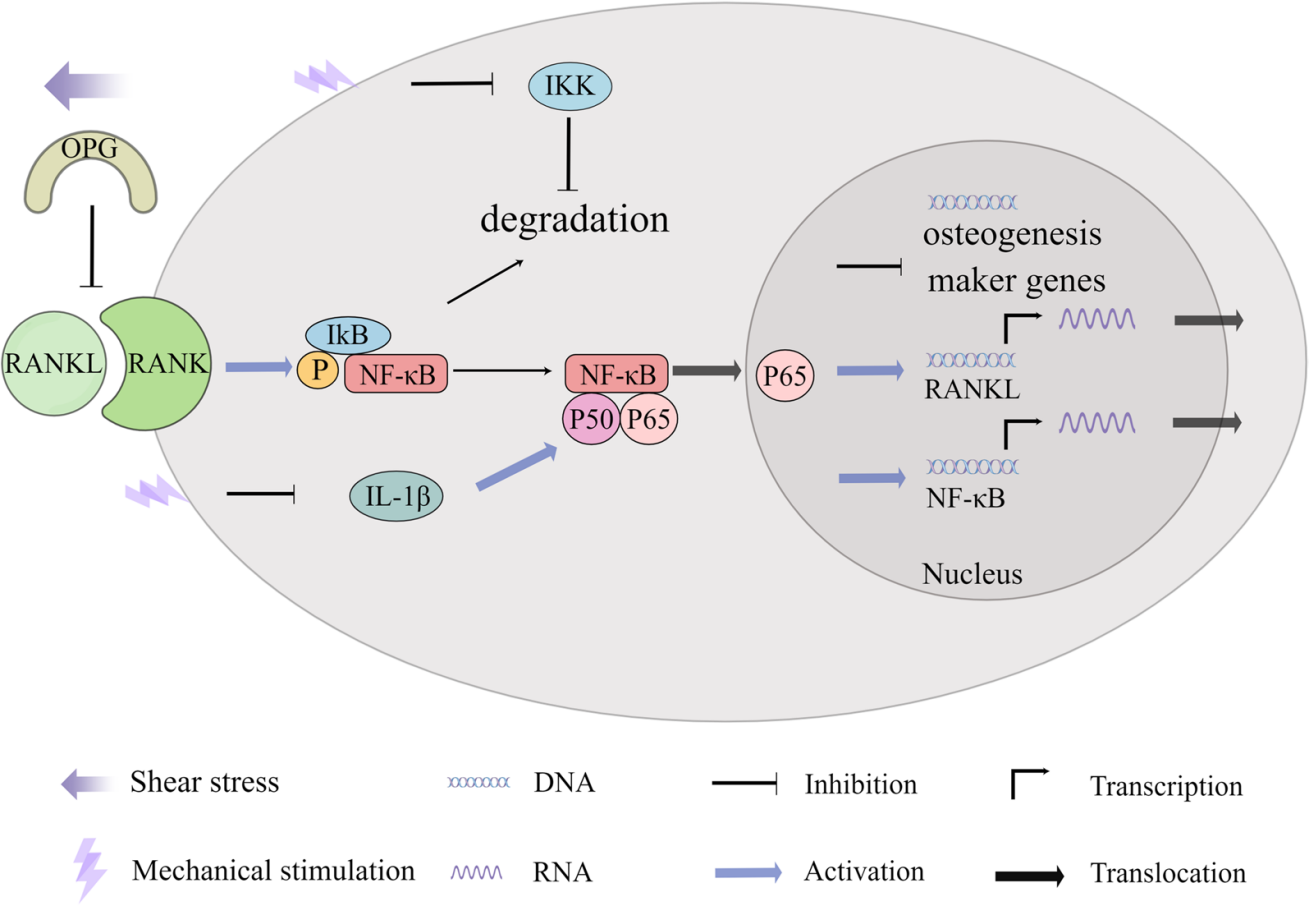
Mechanical stimulation regulates the differentiation of stem cells into osteoblasts/osteoclasts and chondroblasts through the NF-κB pathway. Mechanical stretching can reduce phosphorylated IκB kinase, block NF-κB activity, and promote osteogenic differentiation of cells. Fluid shear stress also increases the expression of OPG, the decoy receptor for RANKL, upregulating the expression of osteoblast marker genes. Mechanical loading can reduce the levels of IL-1β, which in turn reduces NF-κB expression and regulates the chondrogenesis

NF-κB is expressed in rat growth plate chondrogenesis, stimulates chondrocyte proliferation and differentiation, prevents apoptosis, and promotes longitudinal bone growth [85], whereas in hyperchondrogenesis or arthritic cartilage, interleukin (IL)-1β is highly expressed, induces upregulation of miR-381, and promotes cartilage matrix resorption by inhibiting type II collagen and inducing metalloproteinase-13 (MMP-13) [86]. In addition, IL-1β significantly upregulates NF-κB, promotes p65 nuclear translocation, and activates Rac1 and reactive oxygen species (ROS), which in turn activate NF-κB translation in chondrocytes, thereby reshaping the microenvironment for the treatment of ROS and inflammatory factor-related chronic diseases such as osteoarthritis [87]. The increase in miR-320c inhibited cyclin-dependent kinase 6 (CDK6), attenuated IL-1β-induced chondrocyte inflammation, inhibited the activation of NF-kB pathway, and regulated the chondrogenesis of hBMSCs [88]. In contrast, mechanical loading can reduce the levels of oncogenes such as Rac1, MMP9, and IL-1β [89], which in turn reduces NF-κB expression and can modulate the bone microenvironment to reduce the growth and invasion of tumour cells (Fig. 1). Therefore, NF-κB signalling pathway-related proteins may be effective targets for cancer therapy.

Bone formation requires a balance between bone formation by osteoblasts and bone resorption by osteoclasts, the absence of either of which can cause specific corresponding diseases or abnormalities. Therefore, the effects of different mechanical forces on osteoblasts and osteoclasts were investigated. Fluid shear stress reduced RANKL expression and increased OPG expression in cells, which significantly reduced the RANKL/OPG ratio, upregulating the expression of osteoblast marker genes [90] (Fig. 1). Mechanical loading also inhibits osteoclastogenesis and promotes osteogenesis by downregulating NF-κB ligands and receptor activators of histone K, upregulating OPG, and downregulating peroxisome proliferators-activated receptor γ (PPARγ) [89]. However, circulating mechanical strain can stimulate more ALP and calcium deposition through activation of RANKL [91]. Certain circulating stresses can also induce osteoclast differentiation through upregulation of α 7 nAChR and activation of the classical Wnt pathway leading to increased RANKL expression and reduced expression of RUNX2, ALP, and OPG [92]. Exosomes are important mediators in maintaining the balance between bone formation and bone resorption. Exosomes from cyclic mechanical stretch (CMS)-treated BMSCs inhibit actin ring formation and suppress osteoclast differentiation by attenuating the NF-κB signalling pathway, which also provides new insights into intercellular communication between osteoblasts and osteoclasts under mechanical loading [93].

### The nAChR signalling pathway

The nicotinic acetylcholine (ACh) receptor (nAChR) is a ligand-gated ion channel that signals through endogenous ACh and its agonists to drive organoid growth and differentiation [94]. Among these, the α7 nicotinic ACh receptor (α7nAChR) has been the main focus of research [95]. Although most of the research on α7nAChR has involved neural tissue and the inflammatory environment, and less on the stem cell and mechanistic environment, the cholinergic system also involves mammalian non-neuronal cells, such as stem cells. Cholinergic signalling plays a key role in controlling stem cells behaviour [96]. The α7nAChR has also been associated with mechanical signals [92]. In view of this, the α7 nAChR signalling pathway is promising in terms of its relationship to stem cell perception of mechanistic stimuli.

**Fig. 2:**
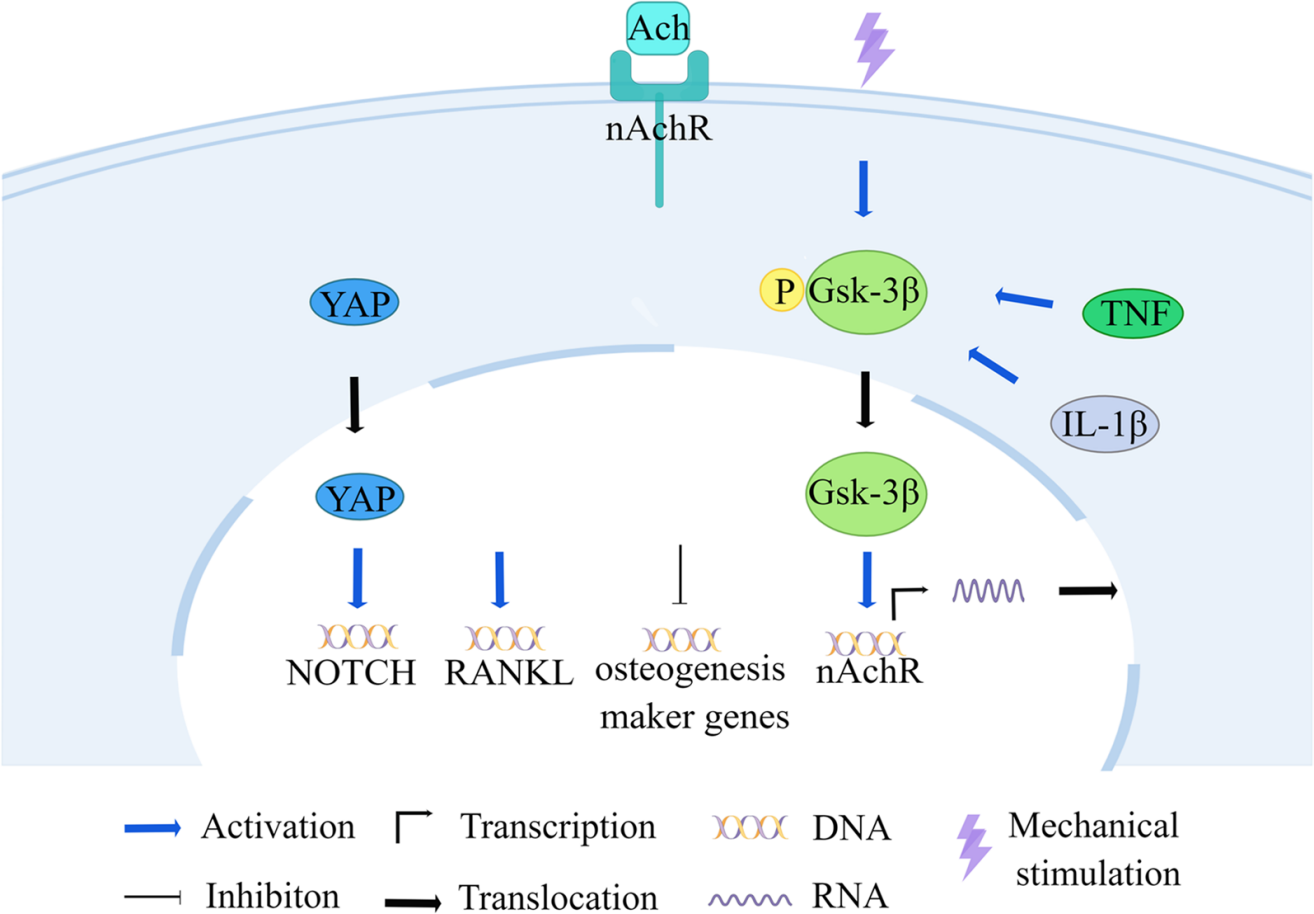
Mechanistic effects attenuate stem cell osteogenic differentiation via the nAchR signalling pathway. Under stress, TNF-α and IL-1β increase phosphorylated GSK-3β in stem cells, which then promotes the expression of α7 nAChR. nAChR is activated by the ligand Ach, which in turn upregulates RANKL and downregulates genes related to osteogenic differentiation

Tumour necrosis factor-α (TNF-α) and IL-1β significantly increased phosphorylated glycogen synthase kinase-3β (GSK-3β) levels in BMSCs and periodontal stem cells (PDLSCs), and increased expression of phosphorylated GSK-3β (p-GSK-3 β) upregulated α7 nAChR expression and promoted its function, which in turn upregulated RANKL, downregulated OPG, and decreased ALP, RUNX2, and OCN, leading to decreased osteogenic differentiation and increased osteoclast formation [97] (Fig. 2). This was also verified in another study in which PDLSCs upregulated the expression of p-GSK-3β, α7nAChR, and active β-catenin and decreased the expression of RUNX2, ALP, and OPG under hydrodynamic stress [92].

At the same time, the nAChR signalling pathway has synergistic effects with a variety of signalling pathways. Upregulation of α7nAChR activates the NF-κB signalling pathway. The receptor activator of RANKL is upregulated, which in turn induces osteoclast effects [92]. The nAChR signal not only coordinates with Wnt signalling to regulate intestinal stem cell (ISC) function [94], but also balances ISC differentiation by activating the Hippo and Notch signalling pathways [98]. The nAChR signalling activator nicotine blocks the osteogenic potential of hPDLC induced by cyclic tensile stress by binding to α7 nAChR and activating the classical Wnt pathway [99]. nAChR signalling activators can also upregulate the downstream effectors of the Hippo and Notch signalling pathways, YAP1/ TAZ and Notch1/Dll1, regulating the expression of target genes [98].

### PIEZO signalling pathway

**Fig. 3:**
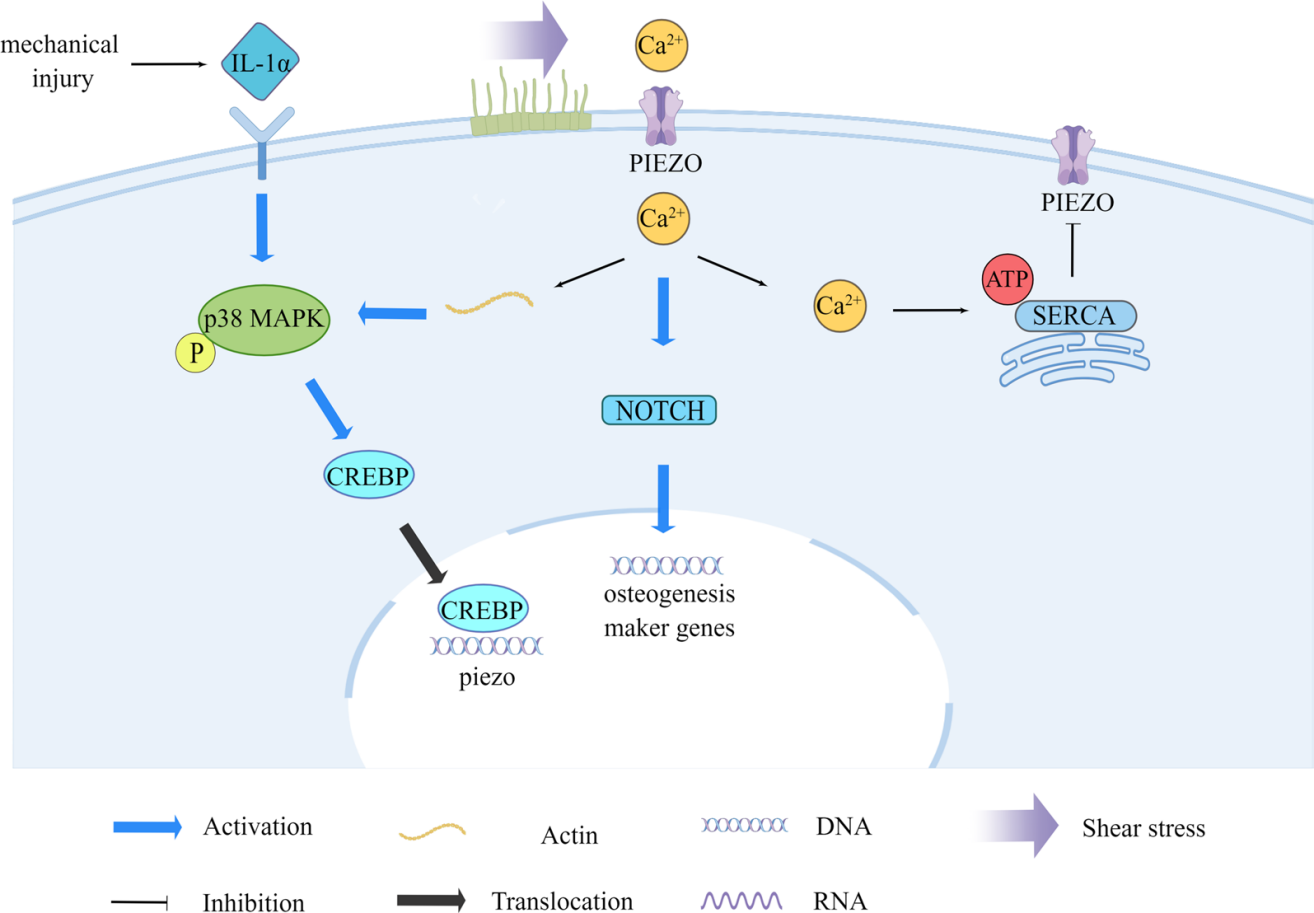
Mechanical stimulation induces osteogenic differentiation of stem cells via the PIEZO pathway. Mechanical stimulation induces cilia, which causes Ca^2+^ to enter the cell via PIEZO, activating the Notch signalling pathway and upregulating osteogenic differentiation genes. Mechanical damage also phosphorylates p38 MAPK via the IL-1α receptor, activating the transcription factor CREBP, which binds to the PIEZO gene promoter and can upregulate PIEZO

The piezoelectric mechanosensitive ion channel (PIEZO) acts as a mechanosensor and is a key receptor for sensing mechanical stimuli [100]. PIEZO strongly controls stem cell differentiation by coordinating WNT expression and ciliogenesis to link mechanical signals to intracellular signals [101]. Mechanical stretching can effectively stimulate osteogenic differentiation of stem cells by activating mechanosensitive ion channels [102] (Fig. 3). In human deciduous dentin stem cells, cyclic stress-induced ciliogenesis and the expression of WNT5b and WNT16, activating PIEZO1 and promoting nuclear translocation of RUNX2, which in turn promoted adult dentin cell differentiation [103]. In addition, stress loading increases PIEZO mRNA expression, which may be related to Ca^2+^ influx [104], which in turn is closely interrelated with cilia. The primary cilia are non-motile cilia and the influx of extracellular Ca^2+^ usually occurs first in the cilia, but the exact mechanism is not known [105]. Increased expression of the PIEZO 1 protein ion channel allows more Ca^2+^ influx into the cell, acting as a second messenger and activating the Notch1 signalling pathway, upregulating the expression of ALP, Runx2, and OCN, thereby promoting the osteogenic differentiation of human periodontal stem cells (hPDLSC) [106].

Shear stress associated with local blood flow is a key piezoelectric channel activator [107], and stimulation of fluid flow in vitro deflects primary cilia on osteocytes, resulting in an immediate rise in cytosolic Ca^2+^. PIEZO 1 responds to shear stress-induced stretching of the cell membrane after mechanical loading, leading to the release of ATP in the extracellular environment. Sarcoplasmic reticulum (SR) Ca^2+^ ATPase 2 (SERCA2) is an ATPase that interacts with PIEZO 1 in the membrane bilayer at the endoplasmic reticulum (ER)-plasma membrane junction and inhibits the mechanosensitivity of PIEZO 1. Specific mechanical forces can also transport cytosolic Ca^2+^ into the SR/ER for storage, maintain Ca^2+^ homeostasis, and regulate the PIEZO pathway [108].

Chondrocytes in articular cartilage are one of the terminal cells of MSC differentiation, and PIEZO channels exhibit a key signal transduction role in the fate of chondrocytes. During endochondral ossification, PIEZO 1 inactivation in chondrocytes impairs trabecular bone formation, resulting in reduced ossification [109]. PIEZO 1 and PIEZO 2 also confer mechanosensitivity to chondrocytes by synergistic action. Mechanical stress induces apoptosis through Ca^2+^ influx from PIEZO to chondrocytes [110] (Fig. 3). PIEZO 2 plays a central role in the apoptotic response to chondrocyte injury [111]. In addition, articular chondrocyte IL-1α receptors can sense mechanical damage and activate transcription factors cyclic AMP (cAMP) response element (CRE)-binding protein 1(CREBP1) by phosphorylating p38 MAPK. CREBP1 binds directly to the proximal PIEZO1 gene promoter and upregulates PIEZO1 expression. PIEZO 1 induces excess intracellular Ca^2+^, and elevated resting-state Ca^2+^ in turn alters the F-actin cytoskeleton and amplifies mechanically induced trauma [112].

### HIF-1α signalling pathway

**Fig. 4:**
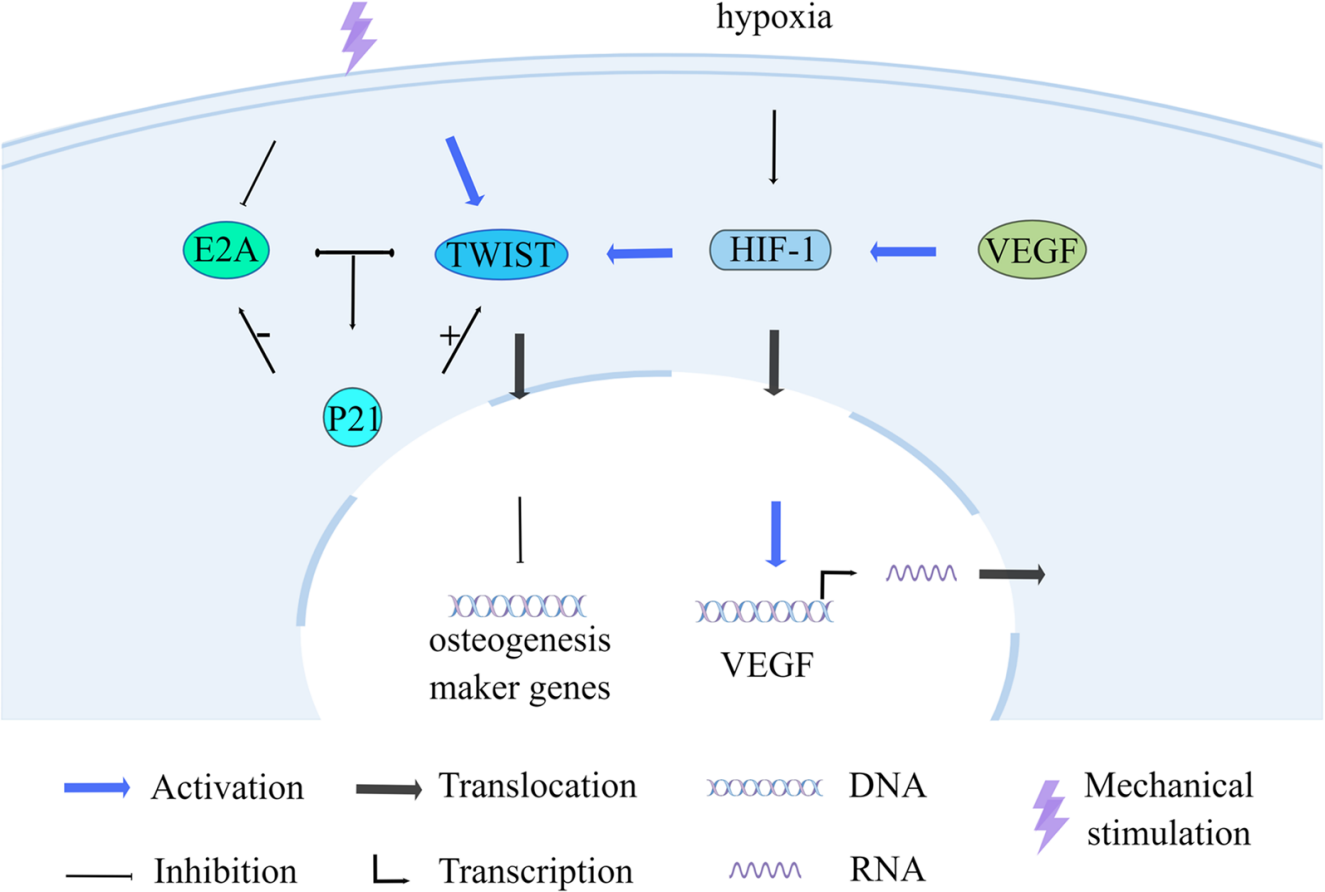
Maintenance of stem cell osteogenic factor homeostasis and maintenance of chondrocyte phenotype through the HIF-1 pathway. HIF-1α increases TWIST expression, which in turn regulates osteogenic differentiation. Mechanical stimulation also promotes TWIST and inhibits E2A; TWIST and E2A interact to activate p21. p21 has different regulatory effects on osteogenic factors. Also, p21 positively regulates the expression of TWIST and negatively regulates the expression of E2A. Hypoxia and HIF-1α maintain the chondrogenic phenotype of cells by preventing cell osteogenic differentiation

Hypoxia-inducible factor 1 (HIF-1) is a basic helix–loop–helix transcription factor that plays a role in apoptosis, and TWIST is also a helix–loop–helix transcription factor that controls gene expression during embryogenesis and the epithelial-mesenchymal transition. The HIF-1α/TWIST-mediated cellular response to oxygen affects stem cell differentiation and bone and cartilage histogenesis [113]. Natural cartilage formation requires hypoxic conditions, whereas bone formation is relatively normoxic [114]. Increased HIF-1α stability under hypoxic conditions stimulates prechondrogenic, anti-bone-forming, and anti-mast cell transcription. At higher oxygen concentrations, HIF-1α degradation promotes hypertrophy and osteoblast formation [115]. Hypoxia and HIF-1α also maintain the chondrogenic phenotype of cells by preventing cell hypertrophy or osteogenic differentiation [116] (Fig. 4). When HIF-1α is conditionally inactivated, the expression of the transcriptional regulator of chondrogenesis SOX9, and its downstream targets is reduced [117].

Under hypoxic conditions, HIF-1ɑ levels in bone marrow macrophages (BMM) are upregulated and can promote osteoclast formation [118]. HIF-1α increases TWIST expression, which in turn decreases RUNX2 and BMP2 expression [119] and regulates BMSC osteogenic differentiation [120]. However, the effect of cyclic tensile stress on Hif-1α expression varies across the stretch range and, in turn, affects different osteogenic differentiation capacities [121]. HIF-1α can also mediate the expression of RUNX2 in PDLSCs through the induction of vascular endothelial growth factor (VEGF) upregulation and promote early osteogenic differentiation [122]. YAP may also be involved in the mechanical stress-induced upregulation of HIF-1α [123].

Mechanical stimulation also induces osteogenic differentiation of BMSCs via the TWIST/E2A/p21 axis. Mechanical cyclic strain promotes TWIST and inhibits E2A, and TWIST and E2A interact to activate p21 expression. p21 has different regulatory effects on RUNX2 and BMP2, maintaining a relative balance between osteogenic factors. Meanwhile, p21 acts as a downstream gene of TWIST and E2A, regulating TWIST expression positively and E2A expression negatively [124] (Fig. 4). HIF-1 can also reduce the differentiation of peripheral blood MSCs (PBMSCs) into osteoblasts by increasing Notch1 expression [125].

## Clinical applications of the mechanical environment of stem cells

### Application of stem cells to treat diseases

Stem cells are receiving increasing attention in the fields of regenerative medicine and tissue engineering and are important for a wide range of diseases [1, 2]. Osteoarthritis, diabetes, osteoporosis, and blood-related diseases are specifically discussed below.

Human adipose mesenchymal progenitor cells (haMPCs) are stem cells with multiple differentiation potentials and immunomodulatory functions. Significant improvements in joint function, pain, quality of life, and cartilage regeneration were observed in patients with knee osteoarthritis after receiving intra-articular injections of ex vivo-expanded haMPCs from their own adipose tissue [126]. MSCs have also shown great potential for differentiation [51] and have applications in the clinic [127]. Injecting MSCs into the joint cavity provided significant relief of osteoarthritis symptoms and no serious adverse effects were observed [128]. Therefore, the application of stem cells in the field of osteoarthritis treatment is of interest. However, further integration and analysis of the efficacy and safety of stem cell therapy are still needed [129], and more research is needed. More types of stem cells are being studied which hold great promise for therapeutic use [130].

Diabetes mellitus (DM) is a serious metabolic disease characterized by hyperglycaemia and beta-cell dysfunction. Although medication is available to control the progression of the disease, it is difficult to cure it, so there is a real need for new and effective treatment modalities for DM. Stem cell therapy holds great promise for people with DM [131]. Stem cell-derived islets are a promising potential treatment for insulin-dependent diabetes [132]. Islet-like organs from progenitor cells are glucose-responsive and insulin-secreting and can reverse disease after transplantation in diabetic mice [133]. Tissue engineering of stem cells derived from adipose tissue can reduce hyperglycaemia and extend lifespan. There is also an opportunity for tissue-engineered islets in future stem cell therapy [33].

Osteoporosis is a widespread progressive bone disease that can pose a serious risk to people’s health and quality of life. Not only does it appear in older adults, but it also commonly afflicts space people who work in outer space. Researchers were inspired by the fact that exercise reduces the risk of osteoporosis in the population. Studies have shown that BMSCs can accelerate bone healing, ossification, and restoration of bone mechanical properties in osteoporotic fractures [134]. BMSCs have therefore become the subject of extensive research. Exosomes from cyclic mechanical stretch (CMS)-treated BMSCs inhibit osteoclastogenesis and ameliorate mechanical unloading-induced bone loss by attenuating NF-κB signalling pathway activity [135]. This result suggests that appropriate mechanical stimulation promotes osteogenic differentiation in BMSCs and provides a theoretical basis for why physical exercise prevents osteoporosis [136].

To overcome the limitations of the small amount of umbilical cord blood stem cells (UCB), intra-bone transfer of UCB (IB-UCB) is used. Intact haematopoietic stem cells were maintained by direct delivery of UCB into hypoxic HSC ecotopes, with rapid haematopoietic recovery and low incidence of graft-versus-host disease [137]. In addition, infusion of umbilical cord MSCs (UC-MSCs) may improve the efficacy of immunosuppressive therapy in children with severe aplastic anaemia and is safe [138]. Also, autologous stem cell transplantation therapy is safe and effective in newly diagnosed multiple myeloma [139]. Stem cells therefore have promising applications in a wide range of blood disorders.

In addition to these common diseases, stem cells have a wide range of applications, including periodontitis [140], acute liver injury [141], liver transplantation [142], and hereditary neonatal hyperammonemia [141], as well as those still waiting to be investigated.

### Regulating the mechanical force of stem cells for disease control

Mechanical signals act as important influences on the fate of living organisms in a number of areas, including the circulatory system [143], neural tissue [144], tendon tissue engineering [145], periodontal tissue engineering [146], cartilage tissue engineering [15], and others. The effects of several mechanical forces on disease are specified below.

The external force generated using an in vitro mechanical device increases the stiffness of adipose tissue, thus affecting the migration and differentiation of ASCs. Different tissue stiffnesses have different effects in promoting the regeneration of adipose tissue. The use of mechanical devices to expand soft tissue holds promise for treating large soft tissue defects that are difficult to reconstruct through surgery [147]. Mechanical modulation of stiffness contributes to the use of MSCs in vascular tissue engineering [44].

Shear stress can cause a variety of clinical conditions. Platelet activation induced by shear stress is thought to be an important mechanism in acute coronary syndromes [148]. Shear stress is also associated with higher white matter lesion volume in migraine patients, which increases with lower endothelial shear stress [149]. Based on the effect of shear stress on disease, corresponding devices have also emerged that have good prospects for application in the medical industry. A fully automated bioreactor system (fABS) enhances the osteogenic differentiation of hBMSCs by generating shear stress on the one hand, and the hypoxia induced by fABS enhances the chondrogenic differentiation of hBMSCs on the other. Thus osteogenesis or chondrogenic differentiation can be balanced by regulating O^2^ concentration and controlling shear stress [47]. Also, a microfluidic dynamic culture system with shear treatment that promotes the expression of blood progenitors by mesenchymal cells and also differentiation towards smooth muscle and cardiomyocytes, acting in the haematopoietic spectrum and arterial vascular system, is promising for the simulation of human embryonic blood formation [45].

Intermittent shear flow has the potential to induce both circumferential stretch caused by HP of the fluid and shear stress caused by flow at the inner surface, while having a role in the simultaneous differentiation of MSC into epithelial and muscular lineages. Intermittent shear flow is more effective than steady shear flow for the development of oesophageal tissue engineering scaffolds [150]. The intermediate filament (IF) network under cyclic HP undergoes disruption and reorganization, translocating towards the perinuclear region, and is a potent mediator of cytoskeletal reorganization and increased osteogenic response in the MSC [59], which also demonstrates a potential new therapy for bone loss diseases such as osteoporosis.

The high HP in the periodontal ligament generated by the orthodontic force recruits the tooth cells and leaves a resorption pit on the root surface. Root resorption is more likely to occur when HP exceeds capillary blood pressure [151]. This finding offers new opportunities to combat orthodontically induced root resorption. In addition, low-intensity vibration therapy as a prophylactic strategy may have the potential as a non-pharmacological alternative to anti-resorptive and anabolic agents without adverse side effects [152], which also offers the possibility of non-pharmacological treatment of degenerative bone disease.

Microgravity has many well-known adverse effects on the human body. Prolonged exposure to microgravity during spaceflight can lead to severe osteoblast dysfunction, resulting in bone loss and causing conditions similar to osteoporosis [72] and disc herniation [153]. In addition, nearly half of the astronauts who landed after a long mission had reduced Hb and developed anaemia, and the magnitude of recovery depended on the duration of space exposure [73]. Astronauts in microgravity also suffer from immune system dysregulation [154] and elevated intracranial pressure (ICP) [155]. With so many adverse symptoms, there is an urgent need to understand the mechanisms by which microgravity causes disease in order to take preventive and remedial measures.

## Conclusions

This paper summarises the impact of mechanical signals, including structural and force signals, in the microenvironment in which stem cells grow on stem cell differentiation and the mechanisms of how stem cells sense mechanical signals, and further discusses how mechanical factors affecting stem cells can be modified for clinical and disease applications. Existing studies on stem cell differentiation mostly ignore the role of mechanical cues in the environment. This review provides a timely summary of the impact of mechanical cues from the microenvironment in which stem cells reside. Mechanical signalling is an integral part of the study of stem cell differentiation that cannot be ignored and provides an important basis for future studies on the specialized differentiation of stem cells. However, the environmental components of stem cell growth are complex, and the mechanical signals generated by the interactions are also complex and diverse [150]. This article only summarises some of the clues, and more mechanistic factors will be discovered in the future. For example, differentiated daughter cells of stem cells are also a component of the stem cell ecotone [156] and daughter cells may also generate some sort of mechanical signal to participate in the composition of the mechanical microenvironment. Additionally, these mechanical cues do not have a single effect on stem cells [157, 158]. And the combination of different mechanical factors is more conducive to the differentiation of stem cells in a more favourable direction [159]. Therefore, how mechanical signals can better cooperate with each other to synergistically influence cell fate should also be considered. In addition, there are a variety of mechanosensing pathways [59, 160, 161], and in future both currently known (but not linked to mechanotransduction) pathways and undiscovered signalling pathways will increasingly be found to play a role in mechanosensing and deserve further investigation. Furthermore, cell membrane tension has an impact on cell fate [162]. Studies have shown that reduced cell membrane tension is a necessary but not sufficient condition for cell fate to shift from self-renewal to differentiation [160]. Therefore, whether cell membrane tension may act as a transmitter in the process of cells sensing the mechanical signals of the extracellular environment and mediate the effects of mechanical changes in the extracellular environment on cell fate deserves further study.

In addition, the performance of implanted stem cells depends not only on differentiation but also on migration, adhesion, proliferation, and paracrine secretion, which deserve to be explored in depth in the future. Different ECM stiffness affects cell-ECM adhesion, cell spreading and migration [163]. The directional migration of cells needs to be guided by certain signals, such as rigidity and topological chemotaxis. Cells have the ability to sense differences in base stiffness and migrate by migrating towards or away from areas of higher stiffness. In addition to this, cells can also sense topographical features of the surrounding environment, known as topological chemotaxis [164]. In addition, genetically reducing the stiffness of the basement membrane in the stratified epidermis increases membrane tension, leading to loss of membrane integrity and enhanced invasiveness of cancerous cells [165]. During tumorigenesis, the ECM undergoes remodelling, which is manifested by changes in molecular composition. The reconstituted ECM exhibits increased tension and stiffness, leading to hyperproliferation, poor differentiation, and invasion and metastasis of tumour cells. When tumour cells grow, the limited space generates compressive mechanical stress, limiting cell proliferation. During epithelial-mesenchymal transition, epithelial cells lose their polarity and cell-to-cell adhesion and acquire the ability to migrate. At the same time, epithelial–mesenchymal transition may promote cell morphological changes and promote proliferation [166]. In addition, different polymers were mixed in any ratio to make microstrip structures and cross-linked into 3D scaffolds to culture MSCs. These mixed microstrips could induce synergy through paracrine signalling to accelerate cartilage regeneration of MSCs [167].

Experiments in which stem cells respond to a mechanical environment sometimes show results that are contrary to previous studies [168], possibly because the cells have a memory of past mechanical cues and this memory remains useful for the behaviour of the stem cells over time [169]. By storing and removing proteins associated with mechanical memory [170], it is possible to alter cell fate, which also provides a direction for stem cell research. The role of stem cell differentiation in medicine is no longer in doubt. In the future, not only will stem cells play a role in the treatment of more diseases, but stem cell-based mechanical microenvironments are expected to be used in a variety of models [77, 171]. Mechanical microenvironments of stem cells will also become increasingly relevant to clinical applications.

In conclusion, this review summarizes the effects of the mechanical microenvironment of stem cell growth on stem cell differentiation and the corresponding mechanisms, which have important implications for clinical disease treatment.

## Data Availability

Not applicable.

## Abbreviations

ECM: Extracellular matrix
dECM: Cell-derived ECM
BMSC: Bone marrow mesenchymal stem cell
β-TCP: β-Tricalcium phosphate
hESC: Human embryonic stem cell
BMP2: Bone morphogenetic protein 2
PCL: Polycaprolactone
ASCs: Adipose stem cells
hASCs: Human adipose stem cells
iPSC: Induced pluripotent stem cell
PLA: Polylactic acid
hMSC: Human mesenchymal stem cell
rAMSCs: Rat adipose mesenchymal stem cells
NSPC: Neural stem progenitor cell
PA: Polyamide
VPCs: Vascular progenitor cells
ECs: Endothelial cells
mMSCs: Mouse mesenchymal stem cells
MSC: Mesenchymal stem cell
hPSC-ECs: Human pluripotent stem cell-derived endothelial cells
rBMSCs: Rat bone marrow mesenchymal stem cells
5-Aza: 5-Azacytidiner
HP: Hydrostatic pressure
PRF: Platelet-rich fibrin
IHP: Intermittent hydrostatic pressure
CHP: Circulating hydrostatic pressure
DBM: Decalcified bone matrix
EPCs: Endothelial progenitor cells
Col I: Type I collagen
GAG: Glycosaminoglycan
Col II: Type II collagen
HSC: Hematopoietic stem cell
MHA/Coll: Magnesium-doped hydroxyapatite/type I collagen composite scaffold
hADSCs: Human adipose-derived stem cells
mESC: Mouse embryonic stem cell
NF-κB: Nuclear factor κ-B
RANK: NF-κB receptor activator
RANKL: Ligand for RANK
RUNX2: Runt-related transcription factor 2
ALP: Alkaline phosphatase
OPG: Osteoprotegerin
IκB: NF-κB inhibitor
IL: Interleukin
ROS: Reactive oxygen species
PPARγ: Peroxisome proliferators-activated receptor γ
CMS: Cyclic mechanical stretch
nAChR: Nicotinic acetylcholine receptor
ACh: Acetylcholine
α7nAChR: α7 Nicotinic acetylcholine receptor
TNF-α: Tumour necrosis factor-α
GSK-3β: Glycogen synthase kinase-3β
PDLSCs: Periodontal stem cells
p-GSK-3 β: Phosphorylated GSK-3β
ISC: Intestinal stem cell
PIEZO: Piezoelectric mechanosensitive ion channel
hPDLSC: Human periodontal stem cell
SR: Sarcoplasmic reticulum
SERCA2: Sarcoplasmic reticulum Ca^2+^ ATPase 2
ER: Endoplasmic reticulum
CREBP1: Cyclic AMP response element-binding protein 1
HIF-1: Hypoxia-inducible factor 1
BMM: Bone marrow macrophage
VEGF: Vascular endothelial growth factor
PBMSCs: Peripheral blood mesenchymal stem cells
haMPCs: Human adipose mesenchymal progenitor cells
DM: Diabetes mellitus
CMS: Cyclic mechanical stretch
UCB: Umbilical cord blood stem cell
IB-UCB: Intra-bone transfer of umbilical cord blood stem cell
UC-MSCs: Umbilical cord mesenchymal stem cells
fABS: Fully automated bioreactor system
IF: Intermediate filament
ICP: Intracranial pressure
MMP-13: Metalloproteinase-13
CDK6: Cyclin-dependent kinase 6
